# Operating region-dependent characteristics of weight updates in synaptic In–Ga–Zn–O thin-film transistors

**DOI:** 10.1038/s41598-022-26123-z

**Published:** 2022-12-12

**Authors:** Danyoung Cha, Yeonsu Kang, Sungsik Lee

**Affiliations:** The Department of Electronics Engineering, Pusan National University, Busan, 46241 Republic of Korea

**Keywords:** Electrical and electronic engineering, Electronic devices

## Abstract

We present a study on characteristics of operating region-dependent weight updates in a synaptic thin-film transistor (Syn-TFT) with an amorphous In–Ga–Zn–O (IGZO) channel layer. For a synaptic behavior (e.g. a memory phenomenon) of the IGZO TFT, a defective oxide (e.g. SiO_2_) is intentionally used for a charge trapping due to programming pulses to the gate terminal. Based on this synaptic behavior, a conductance of the Syn-TFT is modulated depending on the programming pulses, thus weight updates. This weight update characteristics of the Syn-TFT is analyzed in terms of a dynamic ratio (*dr*_*w*_) for two operating regions (i.e. the above-threshold and sub-threshold regimes). Here, the operating region is chosen depending on the level of the gate read-voltage relative to the threshold voltage of the Syn-TFT. To verify these, the static and pulsed characteristics of the fabricated Syn-TFT are monitored experimentally. As experimental results, it is found that the *dr*_*w*_ of the sub-threshold regime is larger compared to the above-threshold regime. In addition, the weight linearity in the sub-threshold regime is observed to be better compared to the above-threshold regime. Since it is expected that either the *dr*_*w*_ or weight linearity can affect performances (e.g. a classification accuracy) of an analog accelerator (AA) constructed with the Syn-TFTs, the AA simulation is performed to check this with a crossbar simulator.

## Introduction

An emerging technology based on non-silicon material has been intensively studied for the futuristic applications, such as a brain-inspired neuromorphic system^1–5^. Here, as a fundamental building block of the neuromorphic system, synaptic devices with the emerging material have been reported^6–10^. For example, thin-film transistors (TFTs) based on an amorphous oxide semiconductors (AOSs), such as In–Ga–Zn–O (IGZO), with a high mobility have been considered as a synaptic device because of their optical and electrical instabilities, mimicking the properties of a biological synapse (e.g. a memory phenomenon).^11–15^. Note that, the optical and electrical instabilities have been believed to be poor properties of the IGZO TFTs for displays^16,17^. However, for the synaptic properties of the IGZO TFTs, electrical instabilities (e.g. defects in the gate oxide) among these are intentionally used. IGZO TFTs with these synaptic properties are conventionally operated at the above-threshold regime due to a relatively high current driving capability^18–21^. This may be expected that the speed of the weight update in the above-threshold regime is faster compared to the sub-threshold regime. However, the above-threshold regime can have a narrow synaptic dynamic-range due to a linear (or parabolic) dependence of the drain current at its regime^22,23^. In addition, for the case of the above-threshold regime, it is expected that the power dissipation is difficult to be decreased because of its relatively high current level^24–26^. On the other hand, the sub-threshold regime of the IGZO TFT can achieve an ultra-low power consumption^27,28^. However, it can be a relatively slower speed of the weight updates due to a lower current level compared to the above-threshold regime, although the speed of the weight updates at the sub-threshold regime can also be reasonable for a neuronal time scale of the human brain which is very long with a range from milliseconds to hundreds of milliseconds^29,30^. Besides these power consumption and speed, it can also be expected that two operating regions are to be different each other in terms of the characteristics of weight updates, such as a dynamic ratio and weight linearity, so it needs to be studied.

In this paper, a study on the characteristics of operating region-dependent weight updates with a synaptic thin-film transistor (Syn-TFT) based on an amorphous IGZO channel layer is presented. In order to achieve a memory function of the IGZO TFT, the defective oxides (e.g. SiO_2_) can be intentionally used for a charge trapping because of programming pulses to the gate terminal (*V*_*GS*_). Based on this memory behavior, a conductance of the Syn-TFT is varied, which is dependent on the programming pulses, thus a weight updates. This characteristics of weight updates is analyzed for two operating regions, such as the above- threshold and sub-threshold regimes, in terms of a dynamic ratio (*dr*_*w*_). Here, the operational regime can be determined depending on the level of the gate read-voltage (${\text{V}}_{\text{GS}}^{\text{ read}}$) relative to the threshold voltage (*V*_*T*_) of the Syn-TFT. To check these, a static and pulsed characteristics of the fabricated Syn-TFT are experimentally monitored using a standard semiconductor analyzer. For the experimental results, it is shown that the *dr*_*w*_ of the sub-threshold regime is larger compared to the above-threshold regime. Moreover, the linear range of the weight in the sub-threshold regime is wider compared to the above-threshold regime. Either the *dr*_*w*_ or weight linearity is expected to affect performances (e.g. a classification accuracy (CA)) of an analog accelerator (AA) where Syn-TFTs are arrayed. To verify this, the AA simulation is performed with a crossbar simulator.

## Syn-TFT and related theories

### Synaptic behavior of the IGZO thin-film transistor

**Figure 1:**
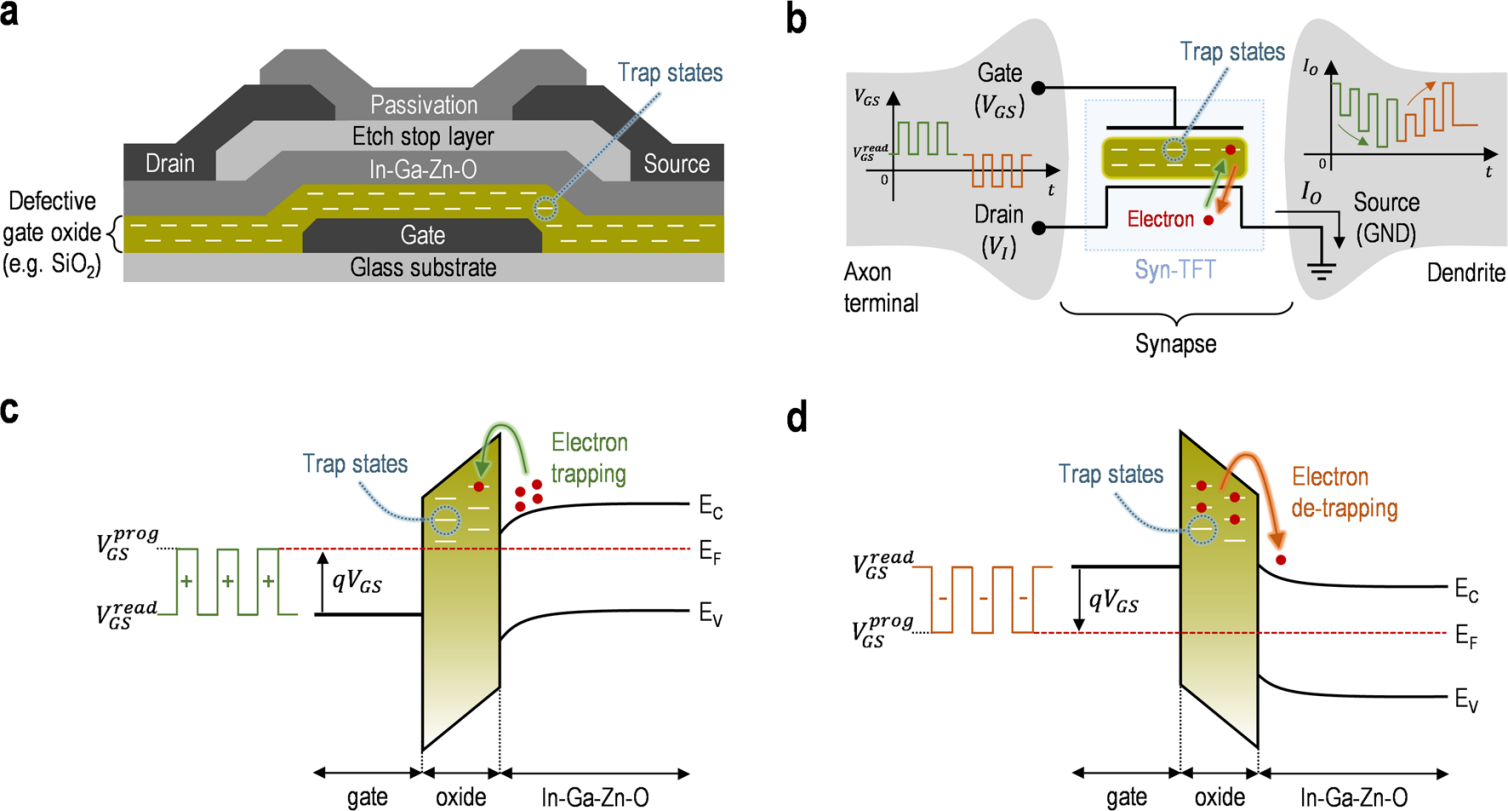
(**a**) Schematic cross-sectional view and (**b**) equivalent circuit of the Syn-TFT. Note that the axon terminal can be considered as the *V*_*GS*_. Band diagrams to describe (**c**) the electron trapping with applying positive programming pulses and (**d**) electron de-trapping with applying negative programming pulses. Here, the *E*_*c*_ is the conduction band minima, *E*_*V*_ is the valence band maxima, and *E*_*F*_ is the Fermi level.

**Figure 2:**
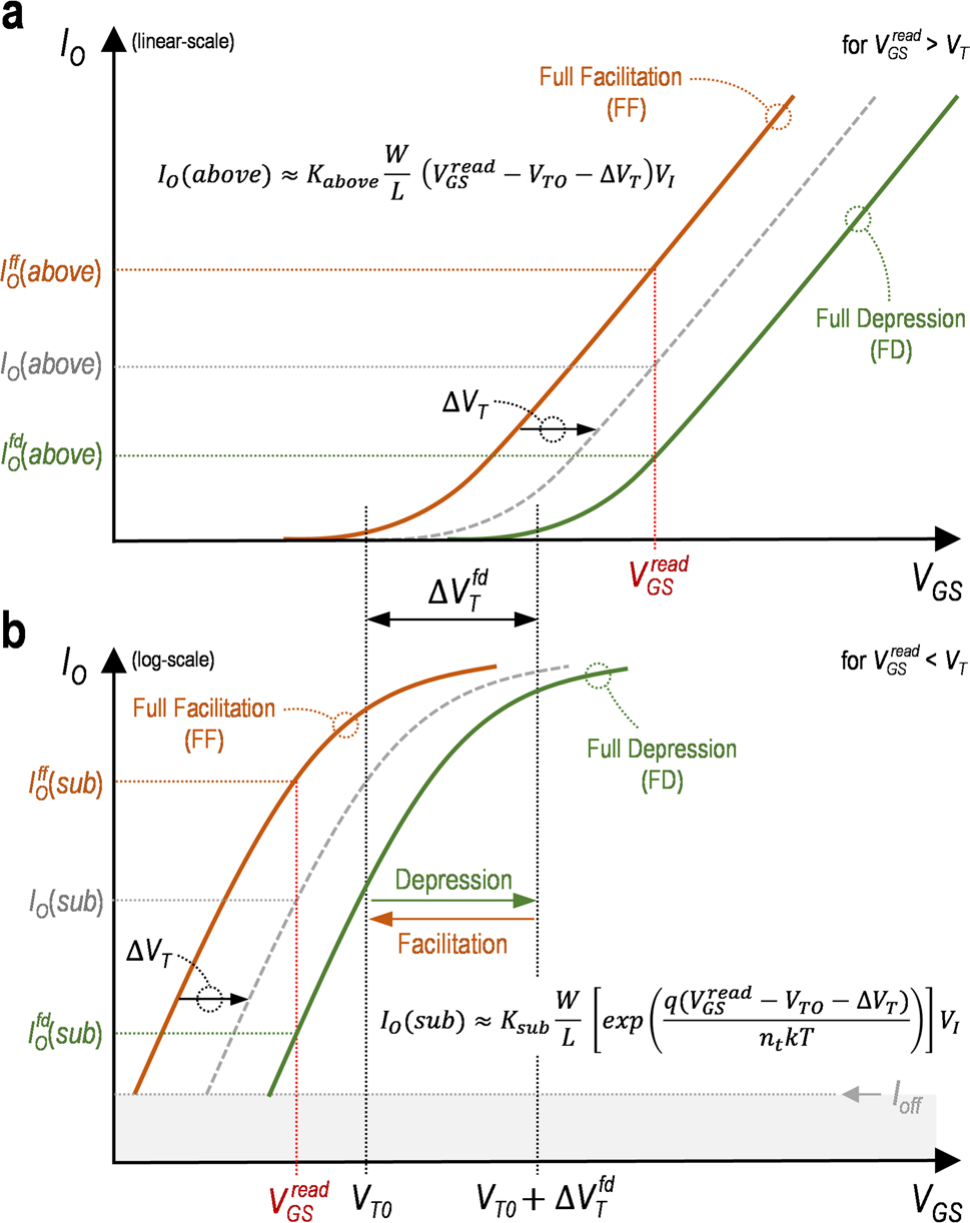
Conceptual plots of the transfer characteristics in (**a**) the above-threshold regime and (**b**) sub-threshold regime for synaptic processes (e.g. the synaptic depression and facilitation) with threshold voltage shifts varied upon programming pulses. Here, the full facilitation and full depression as two extreme states are described for respective operational regime. And the *I*_*O*_(*above*) and *I*_*O*_(*sub*) are shifted toward the full depression with the increase of the $\Delta$*V*_*T*_, respectively (gray dashed lines). In addition, for the case of the sub-threshold regime, the transfer characteristics is depicted as the semi-log plot to clearly show its trend. Note that the *I*_*off*_ is the current off floor.

Figure 1a,b show a cross-sectional view of the Syn-TFT (i.e. the IGZO TFT) and its equivalent circuit, respectively. As can be seen, the defects in the gate oxide (e.g. SiO_2_) of the Syn-TFT can be given due to the disorder of the oxide deposited with a low-temperature process for the TFT^31^. In this defective oxide, multiple trap states can exist, where charges (e.g. electrons) in the IGZO channel layer can be trapped or de-trapped with applying programming pulses to the gate terminal (*V*_*GS*_), so the memory function is achieved. This memory function is depicted in Fig. 1c,d in detail with the band diagrams for the electron trapping and de-trapping, respectively. Here, the electron trapping or de-trapping is dependent on the polarity of the programming voltage (${\text{V}}_{\text{GS}}^{ \, {\text{prog}}}$) and the number of programming pulses applied to the gate for a fixed input voltage (*V*_*I*_) at the drain. Due to these trapped or de-trapped electrons, the threshold voltage shift ($\Delta{\text{V}}_{\text{T}}$) can be varied, which is defined as $\, \Delta{\text{Q}}_{\text{e}}^{\text{trap}}{ /} {\text{C}}_{\text{i}}$. Here, the $\Delta{\text{Q}}_{\text{e}}^{\text{trap}}$ is the variation of the negative charge density per area and *C*_*i*_ is a gate insulator capacitance per area. So, the *V*_*T*_ can also be varied and expressed as follows,

1 $${\text{V}}_{\text{T}}{ = }{\text{V}}_{\text{T0}}{ + \Delta}{\text{V}}_{\text{T}} {,}$$

where the *V*_*T0*_ is an initial value of the *V*_*T*_. For the initial state, assuming that there are no trapped electrons in the gate insulator, $\Delta{\text{V}}_{\text{T}}$ = 0, thus *V*_*T*_ = *V*_*T0*_. And, for the case with applying multiple programming pulses (e.g. positive or negative programming pulses), the $\Delta{\text{V}}_{\text{T}}$ can be varied due to the electron trapping or de-trapping, thus *V*_*T*_ = *V*_*T0*_ + $\Delta{\text{V}}_{\text{T}}$. With this *V*_*T*_, the ${\text{V}}_{\text{GS}}^{ \, {\text{read}}}$ can be set as ${\text{V}}_{\text{GS}}^{ \, {\text{read}}}$ > *V*_*T*_ or ${\text{V}}_{\text{GS}}^{ \, {\text{read}}}$ < *V*_*T*_ to select the operating region (i.e. the above-threshold region or sub-threshold region). Figure 2 describes the schematic transfer characteristics in the above-threshold and sub-threshold regions, respectively. Here, assuming a sufficiently low *V*_*I*_ in two operating regions, the output current (*I*_*O*_) of respective operational regimes is approximated as follows^32^,

2 $${\text{I}}_{\text{O}}\left({\text{above}}\right) \,\approx \, {\text{K}}_{\text{above}} \, \frac{\text{W}}{{\text{L}}} \, \left({\text{V}}_{\text{GS}}^{\text{ read}}\text{ - }{\text{V}}_{\text{T0 }}{- \Delta}{\text{V}}_{\text{T}}\right){\text{V}}_{\text{I}} \, \,\quad \text{for }{\text{V}}_{\text{GS}}^{ \, {\text{read}}}{ > }{\text{V}}_{\text{T}} ,$$

3 $${\text{I}}_{\text{O}}\left({\text{sub}}\right) \,\approx \, {\text{K}}_{\text{sub}}\frac{\text{W}}{{\text{L}}}\left[{\text{exp}}\left(\frac{{\text{q}}\left({\text{V}}_{\text{GS}}^{\text{ read}} -{\text{ V}}_{\text{T0}} {- \Delta}{\text{V}}_{\text{T}}\right)}{{\text{n}}_{\text{t}}{\text{kT}}}\right)\right]{\text{V}}_{\text{I}} \, \,\quad \text{for }{\text{V}}_{\text{GS}}^{ \, {\text{read}}} \, {<} \, {\text{V}}_{\text{T}} ,$$

where the *K*_*above*_ is a constant for the above-threshold regime, *K*_*sub*_ is a constant for the sub-threshold regime, *L* is the channel length, *W* is the channel width, *n*_*t*_ is the ideality factor related to interface states, *kT* is the thermal energy, and *q* is the elementary charge. As seen in Fig. 2, the initial state of the Syn-TFT is a full facilitation (FF) for a maximum value of the *I*_*O*_ (i.e. ${\text{I}}_{\text{O}}^{ \, {\text{ff}}}$) with *V*_*T*_ = *V*_*T0*_ (i.e. $\Delta{\text{V}}_{\text{T}}$ = 0). So, the ${\text{I}}_{\text{O}}^{ \, {\text{ff}}}$ can be rewritten with Eqs. (2) and (3), as follows,

4 $${\text{I}}_{\text{O}}^{ \, {\text{ff}}}\left({\text{above}}\right)\approx{\text{K}}_{\text{above}} \, \frac{\text{W}}{{\text{L}}} \, \left({\text{V}}_{\text{GS}}^{\text{ read}}\text{ - }{\text{V}}_{\text{T0 }}\right) \, {\text{V}}_{\text{I}} \quad \, \, \, {\text{for}} \, {\text{V}}_{\text{GS}}^{\text{ read}}{ > }{\text{V}}_{\text{T},}$$

5 $${\text{I}}_{\text{O}}^{ \, {\text{ff}}}\left({\text{sub}}\right)\approx{\text{K}}_{\text{sub}}\frac{\text{W}}{{\text{L}}}\left[{\text{exp}}\left(\frac{{\text{q}}\left({\text{V}}_{\text{GS}}^{\text{ read}}\text{ } - {\text{ V}}_{\text{T0}} \, \right)}{{\text{n}}_{\text{t}}{\text{kT}}}\right)\right]{\text{V}}_{\text{I}} \quad \, \, \, {\text{for}} \, {\text{V}}_{\text{GS}}^{\text{ read}}{ < }{\text{V}}_{\text{T}},$$

where the ${\text{I}}_{\text{O}}^{ \, {\text{ff}}}\left({\text{above}}\right)$ is the ${\text{I}}_{\text{O}}^{ \, {\text{ff}}}$ for the above-threshold regime and ${\text{I}}_{\text{O}}^{ \, {\text{ff}}}\left({\text{sub}}\right)$ is the ${\text{I}}_{\text{O}}^{ \, {\text{ff}}}$ for the sub-threshold regime.

From the FF, with applying multiple positive programming pulses to the gate, the *I*_*O*_ can be gradually decreased due to the increase of the *V*_*T*_, which is called a synaptic depression process. Here, for the synaptic depression, the increase of the $\Delta{\text{V}}_{\text{T}}$ is limited with applying more programming pulses, thus the saturated $\Delta{\text{V}}_{\text{T}}$. At that moment, the state of the decreased *I*_*O*_ becomes a full depression (FD), defining the maximized $\Delta{\text{V}}_{\text{T}} \, {= \Delta}{{\text{V}} \, }_{\text{T}}^{\text{fd}}$ > 0 (see Fig. 2). Here, the $\Delta{{\text{V}} \, }_{\text{T}}^{\text{fd}}$ is the saturated $\Delta{\text{V}}_{\text{T}}$ with respect to the time although the $\Delta{\text{V}}_{\text{T}}$ can be further enhanced with more programming pulses. Similarly, the *I*_*O*_ for this FD (i.e. ${\text{I}}_{\text{O}}^{ \, {\text{fd}}}$) can be represented with Eqs. (2) and (3), as follows,

6 $${\text{I}}_{\text{O}}^{ \, {\text{fd}}}\left({\text{above}}\right)\approx{\text{K}}_{\text{above}} \, \frac{\text{W}}{{\text{L}}} \, \left({\text{V}}_{\text{GS}}^{\text{ read}}\text{ - }{\text{V}}_{\text{T0 }}{- \Delta}{\text{V}}_{\text{T}}^{ \, {\text{fd}}}\right){\text{V}}_{\text{I}} \quad \, \, \, {\text{for}} \, {\text{V}}_{\text{GS}}^{\text{ read}}\text{ > }{\text{V}}_{\text{T}}{ ,}$$

7 $${\text{I}}_{\text{O}}^{\text{ fd}}\left({\text{sub}}\right)\approx{\text{K}}_{\text{sub}}\frac{\text{W}}{{\text{L}}}\left[{\text{exp}}\left(\frac{{\text{q}}\left({\text{V}}_{\text{GS}}^{\text{ read}} - {\text{ V}}_{\text{T0}}{ - \Delta}{\text{V}}_{\text{T}}^{\text{ fd}}\right)}{{\text{n}}_{\text{t}}{\text{kT}}}\right)\right]{\text{V}}_{\text{I}} \quad \, \, \, {\text{for}} \, {\text{V}}_{\text{GS}}^{\text{ read}}{ < }{\text{V}}_{\text{T}}\text{ .}$$

Here, the ${\text{I}}_{\text{O}}^{ \, {\text{f}}{\text{d}}}\left({\text{above}}\right)$ is the ${\text{I}}_{\text{O}}^{ \, {\text{fd}}}$ for the above-threshold regime and ${\text{I}}_{\text{O}}^{ \, {\text{fd}}}\left({\text{sub}}\right)$ is the ${\text{I}}_{\text{O}}^{ \, {\text{fd}}}$ for the sub-threshold regime. Note that, with applying multiple negative programming pulses from the FD condition, the *I*_*O*_ can be recovered to the initial state (i.e. the FF) because of the decreased *V*_*T*_, which is called a synaptic facilitation process.

### Synaptic weight and dynamic ratio

Based on the synaptic behavior explained in the previous section, the characteristics of weight updates can be theoretically explained for two operating regions. As illustrated in the equivalent circuit of the Syn-TFT (see Fig. 1b), the conductance as a synaptic weight (*G*_*w*_) can be defined as a ratio between the *V*_*I*_ and *I*_*O*_, as follows,

8 $${\text{G}}_{\text{w}} \, \equiv \, \frac{{\text{I}}_{\text{O}}}{{\text{V}}_{\text{I}}} {.}$$

Based on this Eq. (8), the *G*_*w*_ for the FF and FD at ${\text{V}}_{\text{GS}} \, = \, {\text{V}}_{\text{GS}}^{ \, {\text{read}}}$ can be expressed with ${\text{I}}_{\text{O}}^{ \, {\text{ff}}}$ and ${\text{I}}_{\text{O}}^{ \, {\text{fd}}}$, respectively, as follows,

9 $${\text{G}}_{\text{w}}^{ \, {\text{ff}}} \, = \, \frac{{\text{I}}_{\text{O}}^{ \, {\text{ff}}}}{{\text{V}}_{\text{I}}},$$

10 $${\text{G}}_{\text{w}}^{ \, {\text{fd}}} \, =\frac{{\text{I}}_{\text{O}}^{ \, {\text{fd}}}}{{\text{V}}_{\text{I}}},$$

where ${\text{G}}_{\text{w}}^{ \, {\text{ff}}}$ is the *G*_*w*_ for the FF and ${\text{G}}_{\text{w}}^{ \, {\text{fd}}}$ is the *G*_*w*_ for the FD. Here, for a detailed comparison between two operating regions, a normalized synaptic weight ($\overline{{\text{w} }_{\text{G}}}$) needs to be defined. So, using the ${\text{G}}_{\text{w}}^{\text{ ff}}$ as the maximum value of the *G*_*w*_, the $\overline{{\text{w} }_{\text{G}}}$ can be represented with Eqs. (8) and (9), as follows,

11 $$\overline{{\text{w} }_{\text{G}}} \, \equiv \, \frac{{\text{G}}_{\text{w}}}{{\text{G}}_{\text{w}}^{ \, {\text{ff}}}} \, = \, \frac{{\text{I}}_{\text{O}}}{{\text{I}}_{\text{O}}^{ \, {\text{ff}}}}.$$

With Eqs. (2) and (4), the $\overline{{\text{w} }_{\text{G}}}$ in the above-threshold region (i.e. $\overline{{\text{w} }_{\text{G}}}\left({\text{above}}\right)$) can be represented based on Eq. (11), as follows,

12 $$\overline{{\text{w} }_{\text{G}}}\left({\text{above}}\right) \,\approx \, \frac{{\text{V}}_{\text{GS}}^{\text{ read}}\text{ } - {\text{V}}_{\text{TO}}{- \Delta}{\text{V}}_{\text{T}}}{{\text{V}}_{\text{GS}}^{\text{ read}}\text{ } - {\text{V}}_{\text{TO}}} \quad \, \, \, {\text{for}} \, \, \, {\text{V}}_{\text{GS}}^{ \, {\text{read}}}\text{ } > {\text{V}}_{\text{T}}.$$

Similarly, the $\overline{{\text{w} }_{\text{G}}}$ in the sub-threshold region (i.e. $\overline{{\text{w} }_{\text{G}}}\left({\text{sub}}\right)$) can be represented with Eqs. (3) and (5), as follows,

13 $$\overline{{\text{w} }_{\text{G}}}\left({\text{sub}}\right) \,\approx {\text{exp}}\left(\frac{{-{\rm q}\Delta}{\text{V}}_{\text{T}}}{{\text{n}}_{\text{t}}{\text{kT}}}\right) \quad \, \, \, {\text{for}} \, \, \, {\text{V}}_{\text{GS}}^{ \, {\text{read}}} < {\text{V}}_{\text{T}}.$$

Here, the $\overline{{\text{w} }_{\text{G}}}\left({\text{above}}\right)$ is given as a linear function of the $\Delta{\text{V}}_{\text{T}}$ whereas the $\overline{{\text{w} }_{\text{G}}}\left({\text{sub}}\right)$ has an exponential dependence with the $\Delta{\text{V}}_{\text{T}}$. As the two extreme cases of $\overline{{\text{w} }_{\text{G}}}$ commonly for both operating regions, the $\overline{{\text{w} }_{\text{G}}}$ for the FF (i.e. ${\text{I}}_{{\text{O}} \, }{=} \, {\text{I}}_{\text{O}}^{ \, {\text{ff}}}$) and FD (i.e. ${\text{I}}_{\text{O}} \, {=} \, {\text{I}}_{\text{O}}^{ \, {\text{fd}}}$) is expressed based on Eq. (11), respectively, as follows,

14 $$\overline{{\text{w} }_{\text{G}}^{ \, {\text{ff}}}} \, = \, {1},$$

15 $$\overline{{\text{w} }_{\text{G}}^{ \, {\text{fd}}}} \, = \, \frac{{\text{I}}_{\text{O}}^{ \, {\text{fd}}}}{{\text{I}}_{\text{O}}^{ \, {\text{ff}}}},$$

where the $\overline{{\text{w} }_{\text{G}}^{ \, {\text{ff}}}}$ is the $\overline{{\text{w} }_{\text{G}}}$ for the FF and $\overline{{\text{w} }_{\text{G}}^{ \, {\text{fd}}}}$ is the $\overline{{\text{w} }_{\text{G}}}$ for the FD. As can be seen in Eq. (15), it is expected that the $\overline{{\text{w} }_{\text{G}}^{ \, {\text{fd}}}}$ approaches zero since ${\text{I}}_{\text{O}}^{ \, {\text{ff}}}$ > > ${\text{I}}_{\text{O}}^{ \, {\text{fd}}}$.

As another synaptic characteristics, the *dr*_*w*_ can also be defined as a ratio between the $\overline{{\text{w} }_{\text{G}}^{ \, {\text{ff}}}}$ and $\overline{{\text{w} }_{\text{G}}^{ \, {\text{fd}}}}$ with Eqs. (14) and (15), as follows,

16 $${\text{d}}{\text{r}}_{\text{w}} \equiv \, \frac{\overline{{\text{w}}_{\text{G}}^{ \, {\text{ff}}}}}{\overline{{\text{w}}_{\text{G}}^{ \, {\text{fd}}}}}\text{ = }\frac{{\text{I}}_{\text{O}}^{\text{ ff}}}{{\text{I}}_{\text{O}}^{\text{ fd}}}.$$

Note that, for the ideal case, the *dr*_*w*_ is infinity because the $\overline{{\text{w} }_{\text{G}}^{ \, {\text{ff}}}}$ is unity and $\overline{{\text{w} }_{\text{G}}^{ \, {\text{fd}}}}$ is zero, so a large *dr*_*w*_ is advantageous. Likewise, with Eqs. (4) and (6), the *dr*_*w*_ in the above-threshold region (i.e. ${\text{d}}{\text{r}}_{\text{w}}({\text{above}})$) is expressed based on Eq. (16), as follows,

17 $${\text{d}}{\text{r}}_{\text{w}}({\text{above}}){ \approx }\frac{{\text{V}}_{\text{GS}}^{\text{ read}}{ - }{\text{V}}_{\text{TO }}}{{\text{V}}_{\text{GS}}^{\text{ read}}{ - }{\text{V}}_{\text{TO }}{- } \Delta {\text{V}}_{\text{T}}^{ \, {\text{fd}}}} \quad \, \, \, {\text{for}} \, \, \, \, {\text{V}}_{\text{GS}}^{\text{ read}}{ > }{\text{V}}_{\text{T}}.$$

Similarly, the *dr*_*w*_ in the sub-threshold region (i.e. ${\text{d}}{\text{r}}_{\text{w}}({\text{sub}})$) is represented with Eqs. (5) and (7), as follows,

18 $${\text{d}}{\text{r}}_{\text{w}}\left({\text{sub}}\right)\approx{\text{exp}}\left(\frac{{{\rm q}\Delta}{\text{V}}_{\text{T}}^{ \, {\text{fd}}}}{{\text{n}}_{\text{t}}{\text{kT}}}\right) \quad \, \, \, {\text{for}} \, \, \, \, {\text{V}}_{\text{GS}}^{\text{ read}}{ < }{\text{V}}_{\text{T}}.$$

Here, the *dr*_*w*_(*sub*) is expected to be larger compared to the *dr*_*w*_(*above*) because of the exponential dependence in the sub-threshold regime despite of the same $\Delta{\text{V}}_{\text{T}}$ = $\Delta{\text{V}}_{\text{T}}^{ \, {\text{fd}}}$ in two operating regions, implying an advantage of the sub-threshold regime.

Note that, as another advantage of the sub-threshold regime, since the current level in this region is lower compared to the above-threshold regime, it is expected that the static power consumption at ${\text{V}}_{\text{GS}} \, = \, {\text{V}}_{\text{GS}}^{ \, {\text{read}}}$ (${\text{P}}_{\text{static}}^{ \, {\text{read}}}$) is relatively low. The ${\text{P}}_{\text{static}}^{ \, {\text{read}}}$ can be represented for a fixed *V*_*I*_, as follows^33^,

19 $${\text{P}}_{\text{static}}^{ \, {\text{read}}} \, ={ \, {\text{I}}}_{\text{O}}{{\text{V}}}_{\text{I}}.$$

And the ${\text{P}}_{\text{static}}^{ \, {\text{read}}}$(*max*) as a maximum value of the ${\text{P}}_{\text{static}}^{ \, {\text{read}}}$ can be expressed as a product of ${\text{I}}_{\text{O}}^{ \, {\text{ff}}}$ and *V*_*I*_, as follows,

20 $${\text{P}}_{\text{static}}^{\text{ read}}({\text{max}})\text{ } = {\text{ I}}_{\text{O}}^{ \, {\text{ff}}} {\text{V}}_{\text{I}}.$$

Based on the theory with respect to the Syn-TFT, since the static and pulsed characteristics are expected to be different for two operating regions, these characteristics of the fabricated Syn-TFT need to be monitored experimentally.

## Results and discussion

Based on the theoretical analysis in the previous section, it is expected that the synaptic behavior of the IGZO TFT in two operating regions can be achieved due to the trap states in its defective oxide (e.g. SiO_2_), thus the Syn-TFT. The fabrication process of the Syn-TFT is given in following “Materials and fabrication process of the Syn-TFT”^27^. In addition, to verify memory, experimental results of the static and pulsed characteristics of the fabricated Syn-TFT with the IGZO channel layer are described in the following “Selection of the gate read-voltage to determine the operating region” and “Pulsed characteristics of the Syn-TFT”, respectively. For the static characteristics of “Selection of the gate read-voltage to determine the operating region”, the operating region is determined depending on the level of the ${\text{V}}_{\text{GS}}^{ \, {\text{read}}}$ relative to the *V*_*T*_. In “Pulsed characteristics of the Syn-TFT”, the synaptic behavior, such as the weight updates, is analyzed in terms of the *dr*_*w*_ for two operating regions. Here, the experimental framework is performed with a standard semiconductor analyzer, called Keithley™ 4200A. As another observation of the weight updates characteristics, the weight linearity can be analyzed for two operational regimes. In order to check this, the plots of $\Delta\overline{{\text{w} }_{\text{G}}}$ versus $\overline{{\text{w} }_{\text{G}}}$ are shown in the following “Weight linearity of the Syn-TFT”.

In addition, when an array composed of Syn-TFTs is used as an analog accelerator (AA), the performances (e.g. a classification accuracy (CA)) of the AA are expected to be different depending on the operating region. For this, the comparison of the CA as a training result for a classification task is presented in “Analog accelerator based on the Syn-TFT array”. Here, the CA is checked with the AA simulation based on the weight updates monitored with the fabricated Syn-TFT, using a resistive memory simulator, called CrossSim™^34–37^.

### Materials and fabrication process of the Syn-TFT

The fabrication process of the Syn-TFT is as follows. On the glass wafer, RF-sputtering is performed for Molybdenum (Mo) deposition for the gate electrode, assuming that the work-function of Mo is 5 eV^27^. This is followed by wet etching to pattern the gate electrode. And then, SiO_2_ are layered with plasma enhanced chemical vapor deposition (PECVD). The total film thickness of 350 nm is used as the gate insulator where the defects (e.g. trap states) can be given due to a low temperature process. The channel layer of the Syn-TFT is a 50 nm-thick IGZO deposited with RF-sputtering, using an IGZO ceramic target, subsequently patterned by RIE. In the sputter deposition, the oxygen-gas partial pressure against Ar (i.e. O_2_/(O_2_ + Ar)) is 15% to realize Syn-TFT structure, in which each was annealed at 250 ℃. Then an etch-stop layer is formed using SiO_x_ to protect the IGZO channel layer. This is followed by dry etching to define source and drain electrode, respectively. For the final metallization, 150 nm-thick Mo is deposited with a RF sputtering, and patterned with a wet etching. Finally, the device is passivated with a 200 nm-thick SiN_x_/SiO_x_, which followed by another thermal annealing at 250 ℃ for all the samples.

### Selection of the gate read-voltage to determine the operating region

**Figure 3:**
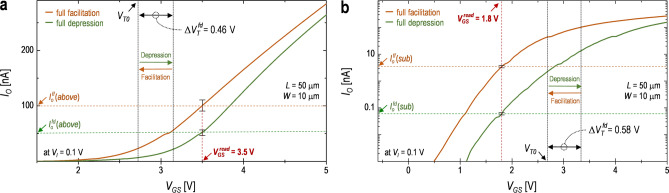
Measured drain current (*I*_*O*_) as a function of the gate voltage (*V*_*GS*_) for the Syn-TFT at the extreme cases [e.g. the full facilitation (FF) and full depression (FD)] for *V*_*I*_ = 0.1 V, where the gate read voltage (${\text{V}}_{\text{GS}}^{\text{ read}}$) is set as (**a**) ${\text{V}}_{\text{GS}}^{\text{ read}}\text{ } > \, {\text{V}}_{\text{T0}}$ in the above-threshold regime and (**b**) ${\text{V}}_{\text{GS}}^{\text{ read}}\text{ } < \, {\text{V}}_{\text{T0}}$ in the sub-threshold regime, respectively. In addition, error bars of 10% are indicated for the *I*_*O*_ at the FF and FD, respectively.

In order to check the characteristics of the weight updates depending on the operating region (i.e. the above-threshold and sub-threshold regions), it is needed to select a proper ${\text{V}}_{\text{GS}}^{ \, {\text{read}}}$. For this, the static characteristics of the fabricated Syn-TFT need to be firstly monitored for the conditions of both ${\text{V}}_{\text{GS}}^{ \, {\text{read}}} > \, {\text{V}}_{\text{T}}$ and ${\text{V}}_{\text{GS}}^{ \, {\text{read}}} \, {<} \, {\text{V}}_{\text{T}}$. Figure 3a,b show the transfer characteristics at the two extreme states (i.e. the FF and FD) for each operational region of the fabricated Syn-TFT, respectively. As can be seen, the ${\text{V}}_{\text{GS}}^{ \, {\text{read}}}$ in the above-threshold and sub-threshold regimes are set as 3.5 V and 1.8 V, respectively. Note that, for the case of ${\text{V}}_{\text{GS}}^{ \, {\text{read}}}$ in the above-threshold regime, since the ${\text{V}}_{\text{GS}}^{ \, {\text{read}}}$ can get into the sub-threshold regime for the shift of the *V*_*T*_ with applying multiple positive programming pulses, a proper ${\text{V}}_{\text{GS}}^{ \, {\text{read}}}$ needs to be chosen satisfying with the condition, i.e. ${\text{V}}_{\text{GS}}^{ \, {\text{read}}}$ > *V*_*TO*_ + $\Delta{\text{V}}_{\text{T}}^{ \, {\text{fd}}}$. For the FF state (i.e. *V*_*T*_ = *V*_*TO*_) with the ${\text{V}}_{\text{GS}}^{ \, {\text{read}}}$ determined in two operating regions, the ${\text{I}}_{\text{O}}^{\text{ ff}}({\text{above}})$ and ${\text{I}}_{\text{O}}^{\text{ ff}}({\text{sub}})$ at *V*_*GS*_ = ${\text{V}}_{\text{GS}}^{ \, {\text{read}}}$ are approximately 100 nA and 3.5 nA, respectively. From this state, after positive programming pulses of 100 cycles are applied to the gate terminal, the *I*_*O*_ in two operational regions at *V*_*GS*_ = ${\text{V}}_{\text{GS}}^{ \, {\text{read}}}$ arrive at the FD state with $\Delta{\text{V}}_{\text{T}}$ = $\Delta{\text{V}}_{\text{T}}^{ \, {\text{fd}}}$ due to multiple electron trapping (see Fig. 1c), resulting in ${\text{I}}_{\text{O}}^{\text{ fd}}({\text{above}})$ = 51 nA and ${\text{I}}_{\text{O}}^{\text{ fd}}({\text{sub}})$ = 0.06 nA, respectively. Here, it is found that the ratio between the ${\text{I}}_{\text{O}}^{ \, {\text{ff}}}({\text{sub}})$ and ${\text{I}}_{\text{O}}^{\text{ fd}}({\text{sub}})$ is larger compared to the ratio between ${\text{I}}_{\text{O}}^{ \, {\text{ff}}}({\text{above}})$ and ${\text{I}}_{\text{O}}^{\text{ fd}}({\text{above}})$, as shown in Fig. 3a,b. This is because the sub-threshold current is dependent on the exponential function, which can be explained with Eqs. (4) to (7). On the other hand, for the case of the above-threshold regime, the current equation is a linear function (see Eq. 2), thus a relatively small ratio between the ${\text{I}}_{\text{O}}^{ \, {\text{ff}}}$ and ${\text{I}}_{\text{O}}^{ \, {\text{fd}}}$. Note that it is found that the $\Delta{\text{V}}_{\text{T}}^{ \, {\text{fd}}}$ of two operating regions, such as the $\Delta{\text{V}}_{\text{T}}^{ \, {\text{fd}}}$ of the above-threshold regime (i.e. $\Delta{\text{V}}_{\text{T}}^{ \, {\text{fd}}}$(*above*) = 0.46 V) and $\Delta{\text{V}}_{\text{T}}^{ \, {\text{fd}}}$ of the sub-threshold regime (i.e. $\Delta{\text{V}}_{\text{T}}^{ \, {\text{fd}}}$(*sub*) = 0.58 V), are to be different each other. This can be explained with a sensitivity (*s*). And the *s* can be represented, as follows^38^,

21 $$\text{s } \, \equiv \, \frac{\Delta{\text{n}}_{\text{e}}}{{\text{n}}_{\text{e}}^{ \, {\text{read}}}},$$

where the $\Delta{\text{n}}_{\text{e}}$ is the negative charge density trapped or de-trapped from the defective gate oxide and ${\text{n}}_{\text{e}}^{ \, {\text{read}}}$ is the negative charge density in the In–Ga–Zn–O film at ${\text{V}}_{\text{GS}} \, = \, {\text{V}}_{\text{GS}}^{ \, {\text{read}}}$. Here, since the ${\text{n}}_{\text{e}}^{ \, {\text{read}}}$ of the sub-threshold regime is smaller compared to the ${\text{n}}_{\text{e}}^{ \, {\text{read}}}$ of the above-threshold regime and the $\Delta{\text{n}}_{\text{e}}$ of the sub- threshold regime can be bigger than the $\Delta{\text{n}}_{\text{e}}$ of the above-threshold regime, the *s* of the sub-threshold regime can be larger compared to the *s* of the above-threshold regime, thus $\Delta{\text{V}}_{\text{T}}^{ \, {\text{fd}}}$(*sub*) > $\Delta{\text{V}}_{\text{T}}^{ \, {\text{fd}}}$(*above*).

Based on the selected ${\text{V}}_{\text{GS}}^{ \, {\text{read}}}$ in respective operating regions, the detailed synaptic processes (i.e. the synaptic depression and facilitation) need to be monitored to verify the characteristics of the weight updates. Therefore, both the detailed pulse-specification and respective pulsed characteristics of the fabricated Syn-TFT are explained in the following section.

### Pulsed characteristics of the Syn-TFT

**Figure 4:**
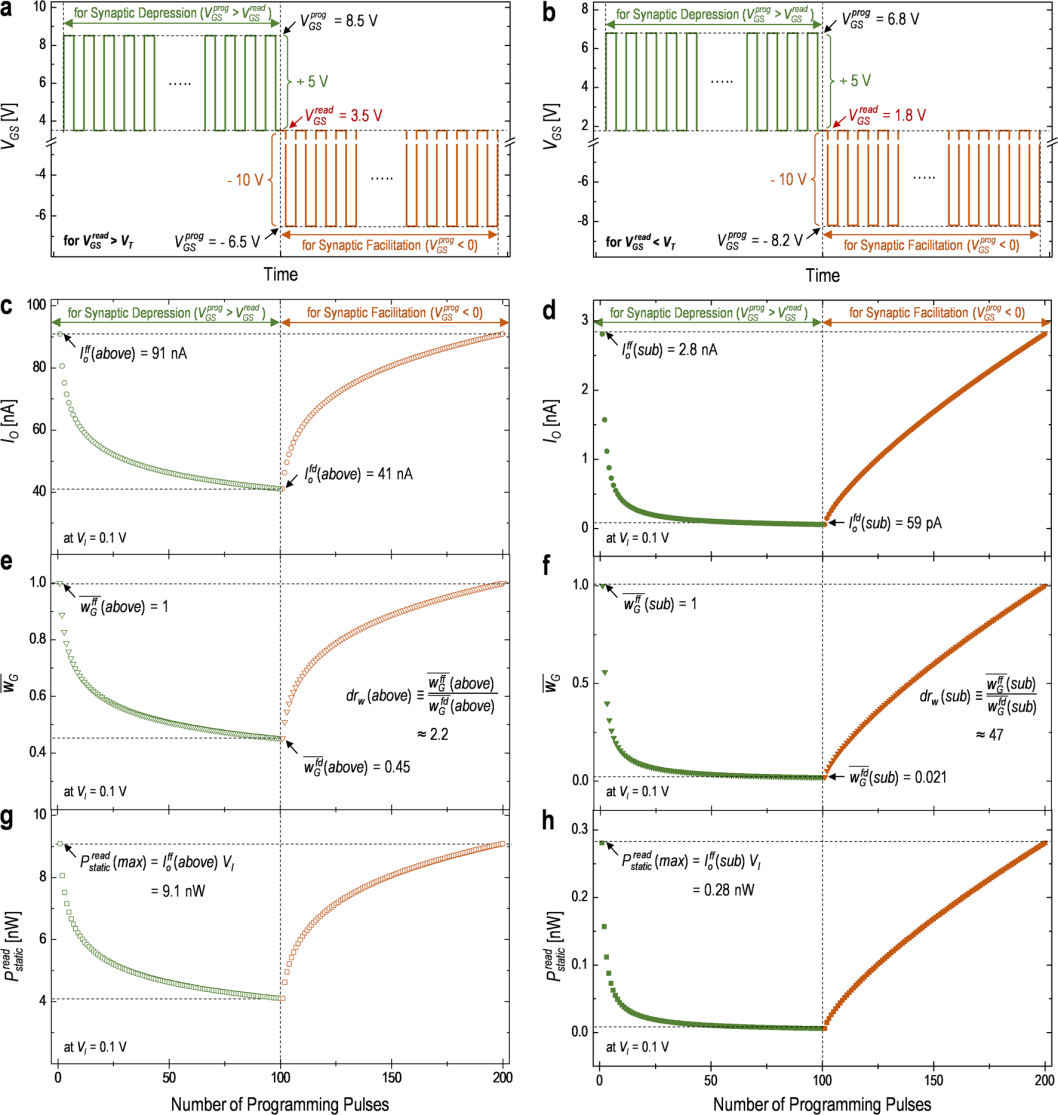
Waveform of the positive/negative programming pulses for the synaptic processes (e.g. a synaptic depression and facilitation) of (**a**) the above-threshold regime and (**b**) sub-threshold regime, respectively. The output current of (**c**) the above-threshold regime and (**d**) sub-threshold regime for the synaptic processes of the Syn-TFT based on the programming pulses, respectively. Here, programming pulses of 100 cycles for respective synaptic process are applied to the gate terminal. The normalized synaptic weight ($\overline{{\text{w} }_{\text{G}}}$) versus the number of the programming pulses for (**e**) the above-threshold regime and (**f**) sub-threshold regime, respectively. Here, the dynamic ratio (*dr*_*w*_) of each operating region can be calculated with the $\overline{{\text{w} }_{\text{G}}}$ of two extreme states. The calculated static-power consumption (${\text{P}}_{\text{static}}^{\text{ read}}$) at the ${\text{V}}_{\text{GS}}^{\text{ read}}$ for the synaptic processes of (**g**) the above-threshold regime and (**h**) sub-threshold regime, respectively, indicating the maximum static power consumption (${\text{P}}_{\text{static}}^{\text{ read}}$(*max*)).

Based on the analysis with respect to the static characteristics of the fabricated Syn-TFT to select the appropriate ${\text{V}}_{\text{GS}}^{ \, {\text{read}}}$ for each operational region, the pulsed characteristics of the device need to be monitored. Figure 4a,b show the pulse-specification for the synaptic depression and facilitation in respective operating regions. For the above-threshold regime, the ${\text{V}}_{\text{GS}}^{ \, {\text{prog}}}$ for the synaptic depression and facilitation is 8.5 V and −6.5 V, respectively, maintaining ${\text{V}}_{\text{GS}}^{ \, {\text{read}}}$ = 3.5 V, as seen in Fig. 4a. For the case of the sub-threshold regime (see Fig. 4b), the ${\text{V}}_{\text{GS}}^{ \, {\text{prog}}}$ is set as 6.8 V and −8.2 V for the synaptic depression and facilitation at ${\text{V}}_{\text{GS}}^{ \, {\text{read}}}$ = 1.8 V, respectively. Note that the pulse height (i.e. the difference between the ${\text{V}}_{\text{GS}}^{ \, {\text{prog}}}$ and ${\text{V}}_{\text{GS}}^{ \, {\text{read}}}$) depending on the synaptic process is commonly given for both operating regions, such as 5 V for the synaptic depression and −10 V for the synaptic facilitation. In addition, the pulse width in two operating regions is commonly set as 3.08 s in the synaptic depression and 37.76 s in the synaptic facilitation, respectively. Other specifications of the programming pulses are common for two operating regions, as follows, the number of cycles and duty cycle for each synaptic process are 100 cycles and 50%, respectively.

With the waveform condition of these programming pulses as seen in Fig. 4a,b, the dynamics of the *I*_*O*_ needs to be firstly monitored for the synaptic depression and facilitation. Figure 4c,d describe the *I*_*O*_ varied for the synaptic processes in two operational regimes. As can be seen, it is found that the *I*_*O*_ is gradually decreased with a series of positive programming pulses for the synaptic depression. It is because the $\Delta{\text{V}}_{\text{T}}$ is increased by electrons trapped into the gate oxide (see Fig. 1c), thus the increase of the *V*_*T*_. This can be explained with Eqs. (2) and (3). After 100 cycles of positive programming pulses for the synaptic depression, the *I*_*O*_ arrives at the FD with $\Delta{\text{V}}_{\text{T}}^{ \, {\text{fd}}}$(*above*) = 0.46 V and $\Delta{\text{V}}_{\text{T}}^{ \, {\text{fd}}}$(*sub*) = 0.58 V, respectively. As a result, the ${\text{I}}_{\text{O}}^{\text{ fd}}({\text{above}})$ and ${\text{I}}_{\text{O}}^{\text{ fd}}({\text{sub}})$ are approximately 41 nA and 0.06 nA in terms of the *I*_*O*_, respectively, as indicated in Fig. 4c,d. During the synaptic facilitation, it is shown that the *I*_*O*_ is gradually increased with applying negative programming pulses because the $\Delta{\text{V}}_{\text{T}}$ is reduced by electrons de-trapped out of the gate oxide (see Fig. 1d), resulting in the recovery of the *V*_*T*_. This can also be explained with Eqs. (2) and (3). And the *I*_*O*_ is almost back to the ${\text{I}}_{\text{O}}^{ \, {\text{ff}}}({\text{above}})$ of 91 nA and ${\text{I}}_{\text{O}}^{ \, {\text{ff}}}({\text{sub}})$ of 2.8 nA, respectively, which corresponds to the FF (see Fig. 4c,d).

Based on the *I*_*O*_ as mentioned earlier, the characteristics of weight updates can be checked and monitored. Figure 4e,f show the behavior of the $\overline{{\text{w} }_{\text{G}}}$ for the synaptic depression and facilitation in two operating regions. During the synaptic depression, it is shown that the trend of the $\overline{{\text{w} }_{\text{G}}}$ decays with applying positive programming pulses due to the increased $\Delta{\text{V}}_{\text{T}}$, which results in the decrease of the *I*_*O*_. Its decay can also be explained with Eqs. (12) and (13). At the FD, the $\overline{{\text{w} }_{\text{G}}}$ in two operational regimes finally approaches at $\overline{{\text{w} }_{\text{G}}^{ \, {\text{fd}}}}$(*above*) = 0.45 and $\overline{{\text{w} }_{\text{G}}^{ \, {\text{fd}}}}$(*sub*) = 0.021, respectively. This is because the *I*_*O*_ arrives at the FD state, which is consistent with Eq. (15). After positive programming pulses continued for 100 cycles, it is found that the $\overline{{\text{w} }_{\text{G}}}$ is facilitated with a series of negative programming pulses, approaching the $\overline{{\text{w} }_{\text{G}}^{ \, {\text{ff}}}}$(*above*) $\cong$ 1 and $\overline{{\text{w} }_{\text{G}}^{ \, {\text{ff}}}}$(*sub*) $\cong$ 1, respectively. It is because of the gradual decrease of the $\Delta{\text{V}}_{\text{T}}$, resulting in the increase of the *I*_*O*_, which can also be explained with Eqs. (12) and (13). In addition, it is found to be a linear increase of the $\overline{{\text{w} }_{\text{G}}}$ for the synaptic facilitation. This can specifically be explained with both the *I*_*O*_ relative to the Δ*V*_*T*_ and the Δ*V*_*T*_ as a function of the number of programming pulses described in Fig. S2 of Supplementary Information.

With both the extracted $\overline{{\text{w} }_{\text{G}}^{ \, {\text{fd}}}}$ and $\overline{{\text{w} }_{\text{G}}^{ \, {\text{ff}}}}$, the *dr*_*w*_ can be calculated as approximately 2.2 in the above-threshold and 47 in the sub-threshold regime, respectively, using Eq. (16). As expected, the *dr*_*w*_(*sub*) is much bigger compared to the *dr*_*w*_(*above*). This is because the exponential function of the sub-threshold regime (see Eq. 18) can be more sensitive compared to the rational function of the above-threshold regime (see Eq. 17) as the $\Delta{\text{V}}_{\text{T}}^{ \, {\text{fd}}}$ for each operating region is comparable (see Fig. 3). Accordingly, for the sub-threshold regime, it is verified that the *dr*_*w*_ is much larger compared to the above-threshold regime. Note that, since *dr*_*w*_(*sub*) > *dr*_*w*_(*above*) with the same pulse width for each synaptic process with respect to two operating regions, it can be considered that the speed of weight updates in the sub-threshold regime is faster compared to the above-threshold regime.

Additionally, as another observation of the pulsed characteristics, the ${\text{P}}_{\text{static}}^{ \, {\text{read}}}$ can be estimated during the synaptic processes for two operational regimes, as seen in Fig. 4g,h. It is found that the ${\text{P}}_{\text{static}}^{ \, {\text{read}}}$ in each operating region decays for the synaptic depression since the *I*_*O*_ is reduced with applying positive programming pulses, which can be explained with Eq. (19). After the synaptic depression, with applying a series of negative programming pulses, it is shown that the ${\text{P}}_{\text{static}}^{ \, {\text{read}}}$ is gradually increased for the synaptic facilitation due to the gradual increase of the *I*_*O*_, which is consistent with Eq. (19). Afterward, at the FF, the ${\text{P}}_{\text{static}}^{ \, {\text{read}}}$ finally arrives at the ${\text{P}}_{\text{static}}^{ \, {\text{read}}}$(*max*) of 9.1 nW in the above-threshold regime and 0.28 nW in the sub-threshold regime, respectively, which can be calculated with Eq. (20). As can be seen, the ${\text{P}}_{\text{static}}^{ \, {\text{read}}}$(*max*) in the sub-threshold regime is found to be about two orders of magnitude smaller than that in the above-threshold regime since the operating current in the sub-threshold regime is much lower compared to the above-threshold regime, as indicated in Fig. 3a,b.

### Weight linearity of the Syn-TFT

**Figure 5:**
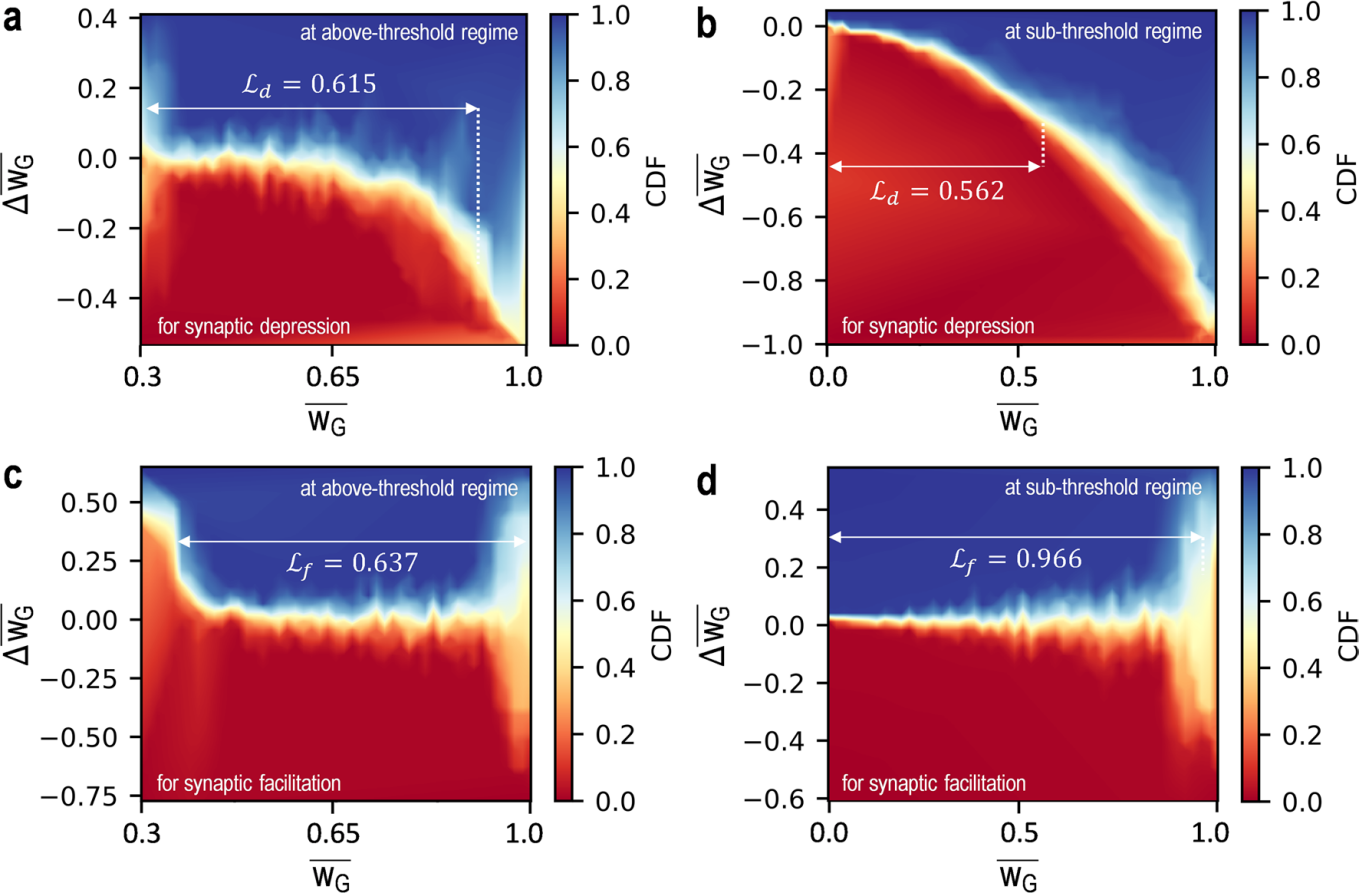
Plots of $\Delta\overline{{\text{w} }_{\text{G}}}$ versus $\overline{{\text{w} }_{\text{G}}}$ of (**a**) the above-threshold regime and (**b**) sub-threshold regime for the synaptic depression, respectively. In addition, for the synaptic facilitation, plots of $\Delta\overline{{\text{w} }_{\text{G}}}$ versus $\overline{{\text{w} }_{\text{G}}}$ of (**c**) the above-threshold regime and (**d**) sub-threshold regime, respectively. For these plots, weight updates of respective operational regime of the Syn-TFT are used and those show the cumulative distribution function (CDF) of the probabilistic variation for the $\Delta\overline{{\text{w} }_{\text{G}}}$. Here, the ${\mathcal{L}}_{d}$ and ${\mathcal{L}}_{f}$ are the linear range of the weight for the synaptic depression and facilitation, respectively. Note that 100 cycles of the programming pulses are applied to the Syn-TFT for the synaptic depression and facilitation of two operating regions, respectively.

As another observation for the characteristics of weight updates (e.g. the *dr*_*w*_), the weight linearity can be checked for two operating regions, using the plots of $\Delta\overline{{\text{w} }_{\text{G}}}$ versus $\overline{{\text{w} }_{\text{G}}}$ based on the extracted data in Fig. 4e,f. Here, the $\Delta\overline{{\text{w} }_{\text{G}}}$ is represented as a difference between the $\overline{{\text{w} }_{\text{G}}^{ \, {\text{n}}}}$ and $\overline{{\text{w} }_{\text{G}}^{ \, {\text{n-1}}}}$ where the $\overline{{\text{w} }_{\text{G}}^{ \, {\text{n}}}}$ is the $\overline{{\text{w} }_{\text{G}}}$ after applying the *n*th programming pulses and $\overline{{\text{w} }_{\text{G}}^{ \, {\text{n-1}}}}$ is the $\overline{{\text{w} }_{\text{G}}}$ before applying the *n*th programming pulse. Figure 5 describes the weight updates of the Syn-TFT with a color map of the cumulative distribution function (CDF) for the synaptic depression and facilitation of those operating regions, respectively, applying the noise components (e.g. the noise power spectral density (NPSD)). Here, the CDF distribution presents a probabilistic variation of the $\Delta\overline{{\text{w} }_{\text{G}}}$ with respect to a synaptic state. In addition, with the plots of $\Delta\overline{{\text{w} }_{\text{G}}}$ versus $\overline{{\text{w} }_{\text{G}}}$, the linear range in the weight update is estimated with 30% variation of the $\Delta\overline{{\text{w} }_{\text{G}}}$. As indicated in Fig. 5a,b, it is shown that the linear range of the weight for the synaptic depression (i.e. ${\mathcal{L}}_{d}$) in the above-threshold and sub-threshold regimes is 0.615 and 0.562, respectively. Moreover, the linear range of the weight for the synaptic facilitation (i.e. ${\mathcal{L}}_{f}$) is found to be 0.637 in the above-threshold regime and 0.966 in the sub-threshold regime, respectively (see Fig. 5c,d). With both the extracted ${\mathcal{L}}_{d}$ and ${\mathcal{L}}_{f}$, the arithmetic mean with respect to the linear range of the weight (i.e. ${\mathcal{L}}_{\text{avg}}=({\mathcal{L}}_{d}+{\mathcal{L}}_{f}) / 2$) for total synaptic processes can be calculated as 0.626 in the above-threshold regime and 0.764 in the sub-threshold regime, thus a good linearity with a wider linear range of the weight in the sub-threshold regime. This can be because a large *dr*_*w*_ as a ratio between the $\overline{{\text{w} }_{\text{G}}^{ \, {\text{fd}}}}$ and $\overline{{\text{w} }_{\text{G}}^{ \, {\text{ff}}}}$ leads to the wide linear range of the weight. In addition, this result is consistent with a linear increase of $\overline{{\text{w} }_{\text{G}}}$ for the synaptic facilitation in the sub-threshold regime, as shown in Fig. 4f. Note that the physical interpretation with respect to the weight linearity is specifically explained with *I*_*O*_ versus $\Delta$ *V*_*T*_ and $\Delta$ *V*_*T*_ versus time (*t*) plots for two operating regions in Supplementary Information (see Fig. S1a–d in Section S1).

Therefore, it is verified that the weight linearity in the sub-threshold regime can be better compared to the above-threshold regime for overall synaptic processes (e.g. the synaptic depression and facilitation), achieving the wide linear range of the weight.

### Analog accelerator based on the Syn-TFT Array

As shown in the previous sections, for the device level, the *dr*_*w*_(*sub*) is found to be larger compared to the *dr*_*w*_(*above*), showing a relatively lower power consumption. And it is also found that the linear range of the weight in the sub-threshold regime is wider compared to the above-threshold regime. These results are summarized in Table 1 for two operating regions.

**Table 1:** Summary of extracted parameters for two operating regions of the fabricated Syn-TFT.

Parameters	Above-threshold regime	Sub-threshold regime
$\Delta{\text{V}}_{\text{T}}^{ \, {\text{fd}}}$ (V)	0.46	0.58
${\text{I}}_{{\text{O}} \, }^{ \, {\text{ff}}}($A)	91 $\times$ 10^–9^	2.8 $\times$ 10^–9^
${\text{I}}_{\text{O}}^{ \, {\text{fd}}}$ (A)	41 $\times$ 10^–9^	6.0 $\times$ 10^–11^
*dr*_*w*_	2.2	47
${\text{P}}_{\text{static}}^{ \, {\text{read}}}({\text{max}})\text{ (}$W)	9.1 $\times$ 10^–9^	28 $\times$ 10^–11^
${\mathcal{L}}_{\text{avg}}$	0.626	0.764

**Figure 6:**
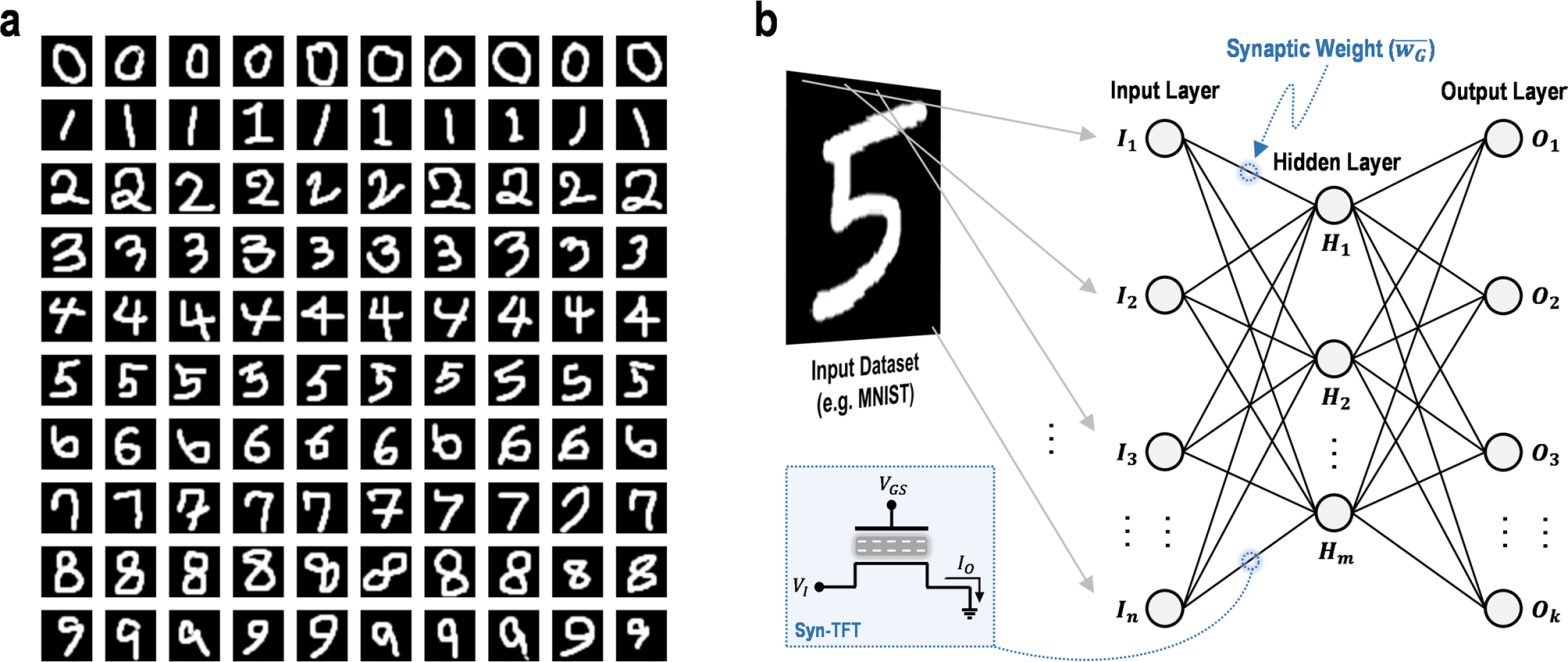
**(a)** Examples of handwritten digits in the MNIST (Modified National Institute of Standards and Technology database) to be classified with an analog accelerator. Here, 28 × 28 grayscale images in the MNIST are used for the training. **(b)** Schematic of an artificial neural network (ANN) with three layers as a simple multi-layer perceptron (MLP) structure. Here, the *I*_*n*_ is *n*th neuron in an input layer, *H*_*m*_ is *m*th neuron in a hidden layer, and *O*_*k*_ is *k*th neuron in an output layer.

**Figure 7:**
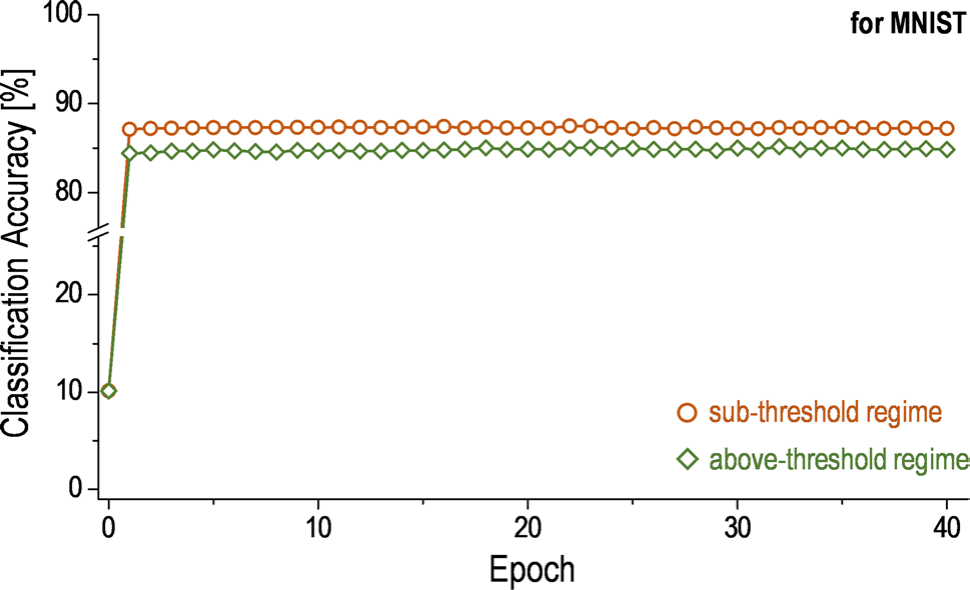
Classification accuracy (CA) of the analog accelerator (AA) based on the Syn-TFT array versus training epochs for the above-threshold regime and sub-threshold regime, respectively. Here, the classification task is performed with the CrossSim™ and tested for the MNIST.

With these results indicated in Table 1, it can be expected that performances, such as the CA, of the AA based on the Syn-TFT array are to be different in those operating regions at the array level. To check this, the CA as a result of the classification task needs to be monitored. Note that handwritten digits in the Modified National Institute of Standards and Technology database (MNIST) are used for the classification task of the AA, as shown in Fig. 6a. To evaluate the performance of the AA based on the Syn-TFT array for an artificial neural network (ANN), the 60,000 training examples and 10,000 test examples are also used (see Fig. 6b)^35^. In addition, the network size, which can be calculated as the product of the size of each layer in the ANN (e.g. the input, hidden, and output layers), is 784 × 300 × 10 for the MNIST classification. Note that, in order to resolve the effects of the *dr*_*w*_ and linearity on the AA performance with respect to two operating regions, the effect of the signal-to-noise ratio (SNR) is intentionally kept the same for each operating region. So, the SNR is equally set in the simulation while considering noise components with 10% of signal for two cases^39^. As a result of the classification task with this AA, the CA is estimated in Fig. 7 for two operating regions, which is tested for 40 epochs. As can be seen, the CA of the sub-threshold regime is found to be bigger compared to the above-threshold regime at each epoch, resulting in the maximum value of 87.51%. This can be explained with the *dr*_*w*_ and ${\mathcal{L}}_{\text{avg}}$ for two operating regions. For the case of the *dr*_*w*_, it is found that the sub-threshold regime is larger than that of the above-threshold regime, as shown in Table 1. Moreover, the ${\mathcal{L}}_{\text{avg}}$ of the sub-threshold regime is found to be wider compared to the above-threshold regime (see Table 1), thus a relatively good weight linearity in the sub-threshold regime. Here, since the SNR, which is proportional to the CA, is intentionally set the same for both two operating regions, it is expected that other parameters, such as the dynamic ratio and weight linearity, would mainly determine the CA. Note that the comparison related to efficiency metrics between proposed Syn-TFT and existing similar synaptic devices is shown in Supplementary Information (see Section S3)^40–43^.

Consequently, these results indicate that the Syn-TFT operated in the sub-threshold regime can achieve a relatively large *dr*_*w*_(*sub*) while showing a relatively wide linear range of the weight. With these results of the device level, when the Syn-TFT array is used as the AA, it is also found that the CA of the sub-threshold regime is larger than that of the above-threshold due to those advantages at the device level of the Syn-TFT operated in the sub-threshold region. Therefore, it is believed that the selection of the sub-threshold regime in the Syn-TFT is essential for a high performance of the ANN while achieving a large *dr*_*w*_ and good weight linearity.

## Conclusion

In this article, an experimental framework on the characteristics of operating region-dependent weight updates for the Syn-TFT with an amorphous IGZO channel layer has been shown. To implement a memory function of the IGZO TFT, the defective oxides, such as SiO_2_, have been intentionally used for a charge trapping or de-trapping because of programming pulses to the gate terminal. Based on this memory function, a conductance of the Syn-TFT is changed, which is dependent on the programming pulses, thus a weight updates. This weight updates characteristics has been analyzed for the above-threshold and sub-threshold regimes in terms of the dynamic ratio, respectively. Here, the operating region has been selected depending on the level of the gate read-voltage relative to the threshold voltage of the Syn-TFT. In order to verify these, a static and pulsed characteristics of the fabricated Syn-TFT have been experimentally monitored with a standard semiconductor analyzer. From the experimental results, it has been shown that the dynamic ratio of the sub-threshold regime ($\approx$ 47) is larger compared to the above-threshold regime ($\approx$ 2.2) and the weight linearity in the sub-threshold regime is better compared to the above-threshold regime. Since either the dynamic ratio or weight linearity is expected to affect performances (e.g. CA) of the AA based on the Syn-TFT array, to check this, the AA simulation has been performed with a crossbar simulator. From the simulation result, it has been found that the CA of the sub-threshold regime is always larger compared to the above-threshold regime at each epoch, indicating the maximum value of 87.51% due to the advantages in the device level of the Syn-TFT operated in the sub-threshold region (e.g. a relatively large dynamic ratio and linear range). Consequently, it can be believed that the proposed Syn-TFT device with both a large dynamic ratio and good linearity for the sub-threshold regime would be essential for the neuromorphic system.

## Supplementary Information

Supplementary Information.

## Data Availability

All data generated or analyzed during this study are included in this published article.
