# Supplementary material for: Operating region-dependent characteristics of weight updates in synaptic In–Ga–Zn–O thin-film transistors
Source: Sci Rep. 2022 Dec 12;12:21441. doi: 10.1038/s41598-022-26123-z (PMC9744913; doi:10.1038/s41598-022-26123-z)
Supplement: Supplementary file 1 — Supplementary Information. [file 41598_2022_26123_MOESM1_ESM.pdf]

# Supplementary Information

# Operating Region-Dependent Characteristics of Weight Updates in Synaptic In-Ga-Zn-O Thin-Film Transistors

Danyoung Cha, Yeonsu Kang, and Sungsik Lee*

Department of Electronics Engineering, Pusan National University, Pusan 46241, Republic of Korea

(*Correspondence to sungsiklee@pusan.ac.kr)

## S1. Physical interpretation with respect to the weight linearity of Syn-TFT

The weight linearity of the fabricated Syn-TFT is found to be poorer in the above-threshold region compared to the sub-threshold region as shown in Section 3-4. As a physical interpretation, this linearity relative to the operating region can be explained with the dependence of the $I_O$ on the time-varying $\Delta V_T$. For this, both the $I_O$ relative to the $\Delta V_T$ and the $\Delta V_T$ as a function of the number of programming pulses for the above-threshold operation and sub-threshold operation are depicted in Figs.S1, respectively. As shown in Figs.S1(a) and (b), the $I_O$ in the above-threshold region is changed linearly with the $\Delta V_T$ whereas that in the sub-threshold region is varied exponentially with the $\Delta V_T$, which is consistent with Eqs.(2) and (3). Moreover, both $\Delta V_T$ in the two operation regions are seemed to be logarithmic functions of the number of programming pulses (see Figs. S1(c) and (d)). So, the $I_O$ in the above threshold is expected to be a composition of a linear and logarithmic functions whereas the $I_O$ in the sub-

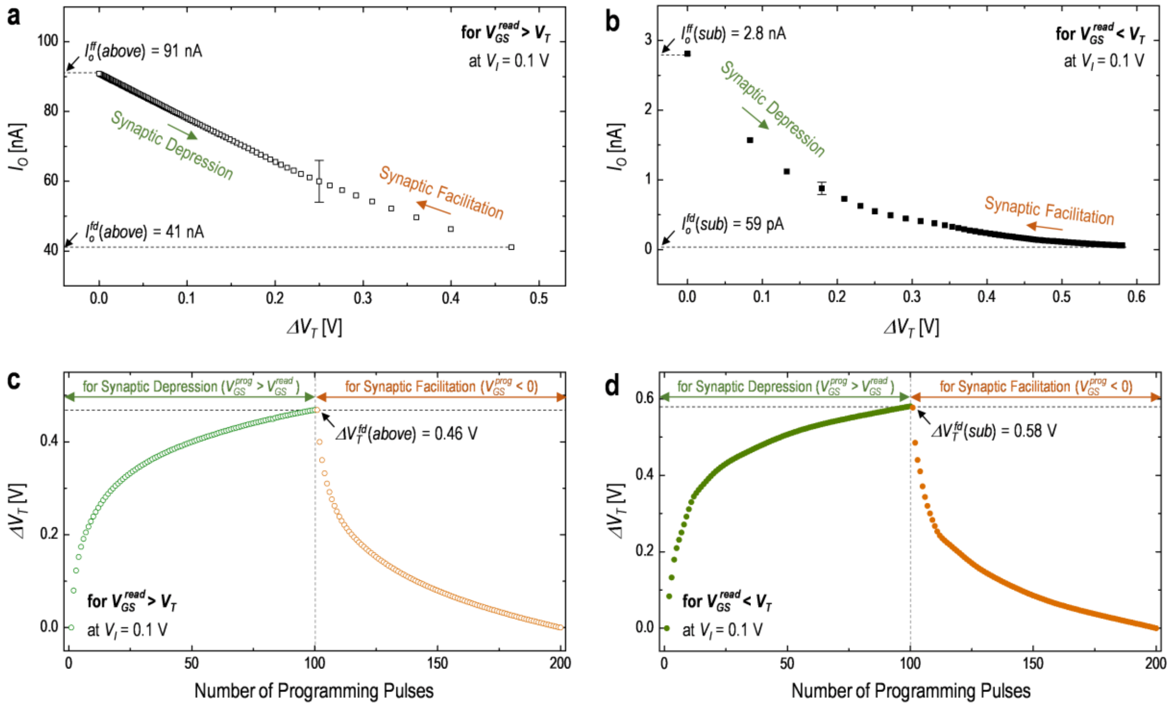


**Figure S1.** Plots of $I_O$ versus $\Delta V_T$ for synaptic processes (e.g. synaptic facilitation and depression) in (a) the above-threshold regime and (b) sub-threshold regime. Here, the error bars of 10 % are illustrated in each plot. In addition, plots of $\Delta V_T$ versus number of programming pulses for the synaptic facilitation and depression in (c) the above-threshold regime and (d) sub-threshold regime.

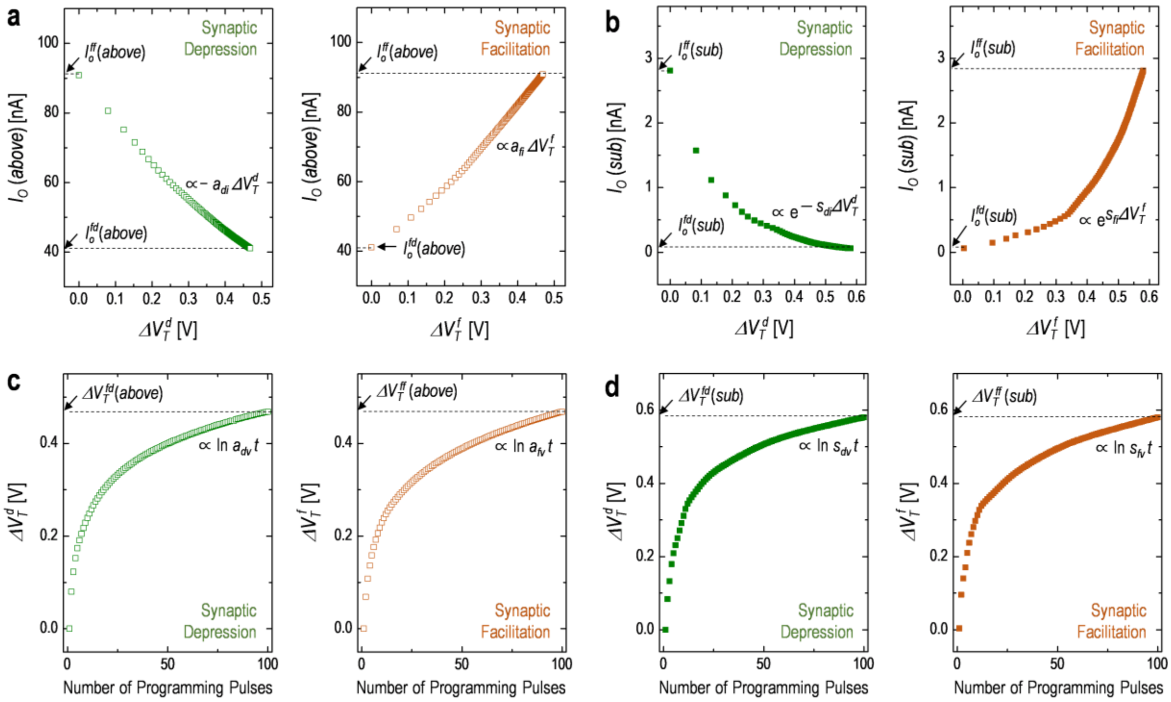

**Figure S2.** The plots of the (a) $I_O$ in the above-threshold regime for the synaptic depression and facilitation, (b) $I_O$ in the sub-threshold regime for the synaptic depression and facilitation redrawn for the $\Delta V_T^d$ and $\Delta V_T^f$. Here, the $I_O(above)$ is proportional to - $a_{di}\,\Delta V_T^d$ or $a_{fi}\,\Delta V_T^f$ for the synaptic depression or facilitation, where the $a_{di}$ and $a_{fi}$ are factors for the linear function of the $\Delta V_T^d$ and $\Delta V_T^f$, respectively. And the $I_O(sub)$ is proportional to $exp$(-$s_{di}\,\Delta V_T^d$) or exp($s_{di}\,\Delta V_T^d$), where the $s_{di}$ and $s_{fi}$ are factors of the exponent in the exponential function of the $\Delta V_T^d$ and $\Delta V_T^f$, respectively. In addition, the $\Delta V_T^d$ and $\Delta V_T^f$ as a function of time in the (c) above-threshold regime and (d) sub-threshold regime, respectively. For the $\Delta V_T^d$, the adv and sdv are factors in the logarithmic function for the above-threshold and sub-threshold regions, respectively. And the $a_{fv}$ and $s_{fv}$ for the $\Delta V_T^f$ are factors in the logarithmic function for the above-threshold and sub-threshold regions, respectively.

threshold region is expected to be a composition of an exponential and logarithmic functions, according to Eqs.(2) and (3). To clarify these relations between the $I_O$ and time-varying $\Delta V_T$, the plots of the $I_O$ and $\Delta V_T$ in Fig.S1 need to be reconstructed with the $\Delta V_T$ separated depending on the synaptic processes (see Fig.S2). When the $\Delta V_T$ in itself for the synaptic depression is defined as $\Delta V_T^d$ and the degree of a recovery in the $\Delta V_T$ for the synaptic facilitation is defined as the $\Delta V_T^f$, both $I_O(above)$ and $I_O(sub)$ of the fabricated Syn-TFT during the synaptic depression appears to be decreased with the $\Delta V_T^d$ whereas those during the synaptic facilitation are increased with the $\Delta V_T^f$ (see Figs.S2(a) and (b)). For these plots of $I_O(above)$ and $I_O(sub)$, the $\Delta V_T^d$ and $\Delta V_T^f$ can be depicted as shown in Figs.S2(c) and (d), respectively.

Here, both $\Delta V_T^d$ and $\Delta V_T^f$ in the two operating regions are found to be proportional to the logarithmic function of time, which is indicated in Figs.S2(c) and (d). In this regard, it can be argued that the time-varying $I_O(above)$ is a composition of the linear function and the logarithmic function for the $\Delta V_T^d$ and $\Delta V_T^f$, which leads to a nonlinear trends of the $I_O(above)$ in Fig.4(c). However, the $I_O(sub)$ can be argued as a composition of the exponential function and logarithmic function for the $\Delta V_T^d$ and $\Delta V_T^f$, as can be seen in Figs.S2(b) and (d), respectively. So, it is probable that the $I_O(sub)$ can be linearly increased with the $\Delta V_T^d$ or $\Delta V_T^f$, which may cause the better linearity of the $I_O(sub)$ shown in Fig.4(d). In addition, the linearity of the $I_O(sub)$ for the synaptic depression is found to be better compared to the synaptic depression (see Fig.4(d)). This may be because the factor of the $I_O(sub)$ for the synaptic facilitation ($s_{fi}$) is closer to unity than that for the synaptic depression ($s_{di}$).

Therefore, due to the region-dependent relations between the $I_O$ and the time-varying $\Delta V_T$, it can be said that the linearity of the $I_O$ can be better for the sub-threshold operation of the Syn-TFT and further enhanced with the factors of the exponent (e.g. $s_{di}$, $s_{fi}$) close to unity. Likewise, since the $w$ is proportional to the $I_O$, the weight update in the above-threshold regime is a nonlinear function. This implies that the weight linearity in the sub-threshold regime can be superior to that in the above-threshold region, resulting in a higher linear range in the sub-threshold region as indicated in Fig.5.

**S2. Solution for the sensing of a low current level of the fabricated Syn-TFT**

When the fabricated Syn-TFT is operated in the sub-threshold regime, a low power consumption can be achieved because of a low current level. However, a low operating current can be expected to be the insufficient current sensing to other circuits (e.g. ADCs) in the next stage. To overcome this, the fabricated Syn-TFT can be connected in series with a resistor to covert the low-level current signal into a voltage signal high enough to be sensed in the

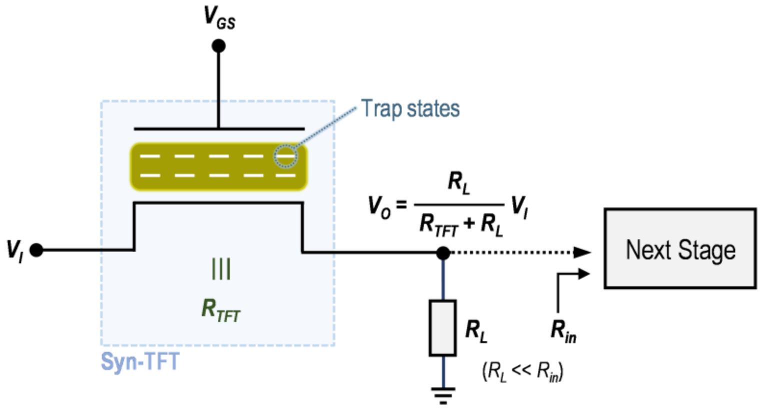


**Figure S3.** Schematic for the Syn-TFT connected in series with a load resistor where the following circuit is connected in parallel. Here, the $R_{TFT}$ is the equivalent resistance of the Syn-TFT, $R_L$ is the load resistance, and $R_{in}$ is the equivalent input impedance of the following circuit. Note that the $R_L$ to be realized in a transistor to tune its value to satisfying $R_L << R_{in}$.

following circuits [4]. Here, the value of load resistor ($R_L$) connected to the Syn-TFT in series is required to be set properly [37]. For example, when a circuit in the next stage is connected in parallel with the load resistor, the input impedance ($R_{in}$) of the circuit needs to be larger compared to the $R_L$ for a sufficient voltage transfer (i.e. $R_{in} >> R_L$), as shown in Fig.S3.

## S3. Comparison between the fabricated Syn-TFT and other synaptic devices

To compare the efficiency metrics between the proposed Syn-TFT and other synaptic devices, figure of merits are summarized in Table S1.

| **Literature** | J. Rao, et al. (2021) [40] | J. Tang, et al. (2018) [41] | N. Mohta, et al. (2021) [42] | Y. Lee et al. (2022) [43] | This paper |
|---|---|---|---|---|---|
| Device type | RRAM | ECRAM | FeS-FET | TFT | TFT |
| Maximum power consumption | 2 μW | 0.03 nW | 125 nW | 70 nW | 0.28 nW |
| Pulse width for weight update | 5 msec | 1 sec | 40 msec | 50 msec | 3.08 sec (depression), 37.76 sec (facilitation) |
| Dynamic ratio | 2.45 | 3 | 12 | 1.62 | 47 |
| Degree of linearity | Poor | Good | Good | Poor | Good (depending on the operating region) |
| Area consumption | 1 μm × 1μm | 80 μm × 100 μm | 2 μm × 4 μm | 150 μm × 500 μm | 50 μm × 10 μm |
| CA (for large images of MNIST) | 88.80% | 96% | 93% | 86.80% | 87.51% |

**Table S1. Summary of figure-of-merits for comparison between other Syn-TFT and synaptic devices.**
